# A cuproptosis random forest cox score model-based evaluation of prognosis, mutation characterization, immune infiltration, and drug sensitivity in hepatocellular carcinoma

**DOI:** 10.3389/fimmu.2023.1146411

**Published:** 2023-03-30

**Authors:** Ruiqi Liu, Yingyi Liu, Fengyue Zhang, Jinrui Wei, Lichuan Wu

**Affiliations:** ^1^ School of Medicine, Guangxi University, Nanning, China; ^2^ Guangxi Scientific Research Center of Traditional Chinese Medicine, Guangxi University of Chinese Medicine, Nanning, Guangxi, China

**Keywords:** cuproptosis, hepatocellular carcinoma, prognostic signature, immunotherapy, tumor microenvironment

## Abstract

**Background:**

Hepatocellular carcinoma is the third most deadly malignant tumor in the world with a poor prognosis. Although immunotherapy represents a promising therapeutic approach for HCC, the overall response rate of HCC patients to immunotherapy is less than 30%. Therefore, it is of great significance to explore prognostic factors and investigate the associated tumor immune microenvironment features.

**Methods:**

By analyzing RNA-seq data of the TCGA-LIHC cohort, the set of cuproptosis related genes was extracted *via* correlation analysis as a generalization feature. Then, a random forest cox prognostic model was constructed and the cuproptosis random forest cox score was built by random forest feature filtering and univariate multivariate cox regression analysis. Subsequently, the prognosis prediction of CRFCS was evaluated *via* analyzing data of independent cohorts from GEO and ICGC by using KM and ROC methods. Moreover, mutation characterization, immune cell infiltration, immune evasion, and drug sensitivity of CRFCS in HCC were assessed.

**Results:**

A cuproptosis random forest cox score was built based on a generalization feature of four cuproptosis related genes. Patients in the high CRFCS group exhibited a lower overall survival. Univariate multivariate Cox regression analysis validated CRFCS as an independent prognostic indicator. ROC analysis revealed that CRFCS was a good predictor of HCC (AUC =0.82). Mutation analysis manifested that microsatellite instability (MSI) was significantly increased in the high CRFCS group. Meanwhile, tumor microenvironment analysis showed that the high CRFCS group displayed much more immune cell infiltration compared with the low CRFCS group. The immune escape assessment analysis demonstrated that the high CRFCS group displayed a decreased TIDE score indicating a lower immune escape probability in the high CRFCS group compared with the low CRFCS group. Interestingly, immune checkpoints were highly expressed in the high CRFCS group. Drug sensitivity analysis revealed that HCC patients from the high CRFCS group had a lower IC_50_ of sorafenib than that from the low CRFCS group.

**Conclusions:**

In this study, we constructed a cuproptosis random forest cox score (CRFCS) model. CRFCS was revealed to be a potential independent prognostic indicator of HCC and high CRFCS samples showed a poor prognosis. Interestingly, CRFCS were correlated with TME characteristics as well as clinical treatment efficacy. Importantly, compared with the low CRFCS group, the high CRFCS group may benefit from immunotherapy and sorafenib treatment.

## 1 Introduction

Liver cancer remains one of the most lethal cancers, with 830,000 deaths worldwide in 2020, accounting for 8.3% of cancer related deaths (1). Hepatocellular carcinoma (HCC) is the most frequent of all primary liver cancers, comprising 75-85% of cases (2). Due to the lack of diagnostic marker, most of the HCC patients are diagnosed at advanced stages with a poor prognosis (3). Therapies such as traditional cytotoxic drugs are rarely effective. Over the last decade, sorafenib and lenvatinib are the only systemic drugs that have been proven to be clinically effective in the therapy of part of the advanced HCC patients (4). Therefore, it is crucial to find valid prognostic models as well as treatment strategies.

Immune checkpoint inhibitor (ICIs) therapy is one of the fastest-developing immunotherapy strategies, which effectively breaks the dilemma of cancer treatment, especially in advanced cancer. However, the efficacy of immunotherapy varies widely among patients (5). HCC is intimately correlated with inflammation and has a complicated tumor microenvironment (TME) (6). Immune checkpoint therapy is being used for HCC treatment recently. The sensitivity of immunotherapy in HCC varies significantly due to the heterogeneity and complexity of the TME (7). Revealing the potential TME characteristics of HCC patients is hence crucial for predicting the efficacy of immunotherapy.

Copper (Cu) is a required element for human health. Disturbance of intracellular coppers is associated with diverse pathologies (8). Previous studies have demonstrated that Cu levels are significantly increased in tumor tissues and cancer patients derived serum (9–12). The elevated levels of Cu are reported to be involved in tumor cell proliferation, angiogenesis, and metastasis (13, 14). Cu may also increase the incidence of HCC in Wilson’s disease patients (15). Both copper chelators and copper ionophores have been exploited as antitumor drugs and tested in clinical trials (16–18). Besides, Cu homeostasis is essential for maintaining normal immune function (19–21) and elevated Cu levels in tumor cells contribute to immune escape by enhancing PD-L1 expression (22). These findings suggest that Cu plays an important role in tumorigenesis and TME shaping. The Cu metabolism is recognized as a unique vulnerability in cancer (23) and targeting Cu metabolism might be an alternative strategy for cancer treatment (24). Recently, a novel Cu induced programmed cell death termed cuproptosis was revealed which occurs by targeting lipoylated TCA cycle proteins (25). Previous studies have shown that cuproptosis-related signature and genes are closely related to TME in colorectal cancer (26), breast cancer (27), lung cancer (28), bladder cancer (29), kidney renal clear cancer (30), and so forth. However, the relationships between cuproptosis-related genes and prognosis, immune microenvironment, and drug sensitivity of liver cancer has not been fully elucidated.

In this study, cuproptosis-related gene sets were derived by correlation analysis as generalization features. Then a random forest Cox prognostic model was constructed, and the cuproptosis random forest Cox score (CRFCS) was built by random forest feature filtering and univariate multivariate Cox regression. The HCC patients were clustered according to CRFCS and investigated in terms of prognosis analysis, mutational characteristics, tumor microenvironment, prediction of immune evasion, immune checkpoint, and drug sensitivity.

## 2 Materials and methods

### 2.1 Data acquisition and processing

The mRNA expression data, somatic mutation data, and corresponding clinical information of HCC were downloaded from the TCGA database *via* the R package “TCGAbiolinks”. The clinical and mRNA expression data of GSE116174 and ICGC-LIHC-US cohorts were downloaded from the GEO database (https://www.ncbi.nlm.nih.gov/geo/) and the ICGC database (https://dcc.icgc.org/projects/), respectively. Then, the mRNA data were converted to TPM format and normalized by log2 transformation.

### 2.2 Development of cuproptosis random forest cox score (CRFCS)

The cuproptosis-associated gene set was derived as a generalization feature by correlation analysis based on the TCGA-LIHC cohort. We used the method “rfsrc” in the R package “randomForestSRC” to construct a random forest model and selected features. The Cox regression was constructed based on the mentioned characteristics, and Regression coefficients were obtained by the “coxph” method in the “survival” package. The Cuproptosis Random Forest Cox Score (CRFCS) was established by the following formula:


$$Score=\sum^{\;}E_{i}r_{i}$$

Where $E_{i}$ is the expression of feature gene i, and $r_{i}$ is the characteristic co-efficient of feature gene i.

### 2.3 Survival analysis

Kaplan-Meier (K-M) survival analysis and visualization were conducted with the “survival” and “survminer” packages. The time-related receiver operating characteristic curve (time ROC) was performed by the R package “pROC” to evaluate the prediction performance of CRFCS in the training and test sets.

### 2.4 Processing and analysis of mutation profile

The analysis and visualization of mutation profile were performed by the “maftools” package. We plotted the mutation waterfall by the method “oncoplot”. After removing the loci falling into the CNV region, the Mutant-Allele Tumor Heterogeneity (MATH) score of the samples was calculated by the “inferHeterogeneity” method (31). MSI scores were calculated by the “MSIsensor” method (32).

### 2.5 TME cell infiltration assessment

The immune cell infiltration was estimated by both ssGSEA and CIBERSORT algorithms. For the ssGSEA method, we used the TME-infiltrating gene set from Charoentong et al., which includes 28 immune cell types (33). We evaluated the enrichment fraction of each sample in the cohort *via* the ssGSEA method to characterize the immune cell invasion in each sample. The CIBERSORT algorithm worked in conjunction with the immune infiltration signature matrix LM22 to evaluate the invasion of various immune cells in the samples. In the case of stromal cells, we estimated the stromal cell infiltration by evaluating the expression of markers for each stromal cell.

### 2.6 Immune evasion prediction

The Tumor Immune Dysfunction and Exclusion (TIDE) algorithm is used to assess the immune evasion mechanism of tumors (34). The effect of both T-cell dysfunction and T-cell exclusion mechanisms on immune evasion was evaluated separately by the TIDE algorithm and the TIDE score was used to predict the degree of immune evasion of the samples.

### 2.7 Drugs sensitivity prediction

The IC_50_ values of the drugs in the training set samples were evaluated by the “pRRopheticPredict” method of the R package “pRRophetic”, with the dataset “cgp2016”. We calculated the correlation between IC_50_ values and CRFCS subgroups to investigate the association between CRFCS and drug sensitivity.

### 2.8 Statistical analysis

The analysis and visualization of the data were performed in R (version 4.1.1). The Wilcoxon test was used to compare the data between the two groups. Charts were mainly visualized by the “ ggplot2 “ package. The p-value<0.05 was regarded as statistically significant (*p<0.05; **p<0.01; ***p<0.001; ****p<0.0001).

## 3 Results

### 3.1 The expressions and prognosis analysis of cuproptosis-related genes in HCC

**Figure 1:**
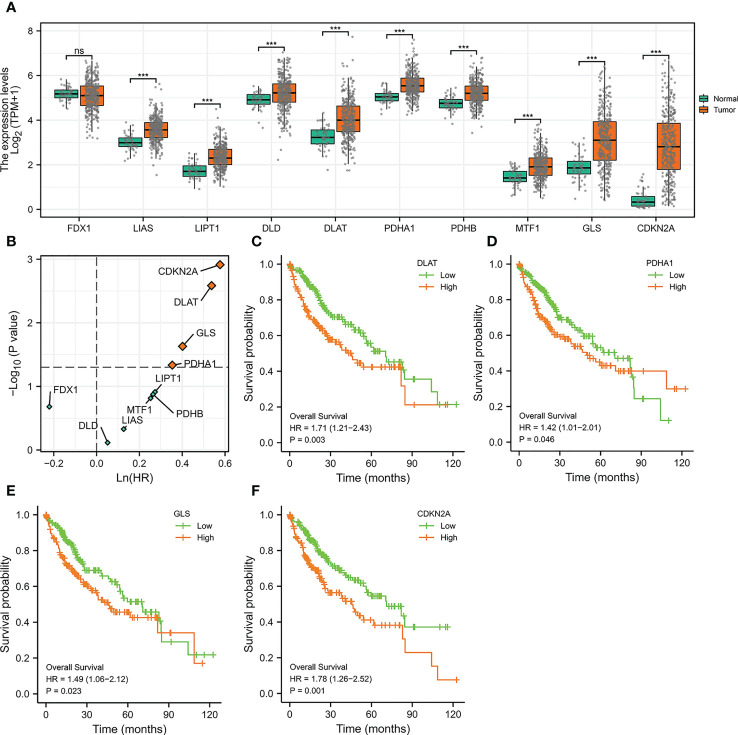
The Expressions and Prognosis Analysis of Cuproptosis-Related Genes in HCC. **(A)** Differential expression of cuproptosis-related genes in the TCGA-LIHC cohort. (***p<0.001; ns stands for not significant) **(B)** Correlation between cuproptosis-related gene expression and survival data (OS) of HCC patients. The horizontal dotted line stands for p=0.05. The vertical dotted line represents HR=1. **(C–F)** Kaplan-Meier curves of DLAT **(C)**, PDHA1 **(D)**, GLS **(E)**, and CDKN2A **(F)**.

We initially evaluated the expressions of ten genes in HCC which were reported to be crucial regulators of cuproptosis (25). It was noticed that among these ten genes, all of them except FDX1 were significantly highly expressed in HCC (**Figure 1A**), indicating that the cuproptosis process might be associated with HCC. To further explore the prognosis of cuproptosis genes in HCC, we performed a correlation analysis between cuproptosis gene expression and HCC patients’ survival (OS) (**Figure 1B**). The results displayed that genes DLAT (HR =1.71, p =0.003), PDHA1 (HR =1.42, p =0.046), GLS (HR =1.49, p =0.023), and CDKN2A (HR =1.78, p =0.001) had prominent prognostic significance in HCC, and patients with high expression of these four genes exhibited shorter survival (**Figures 1C–F**).

### 3.2 Construction of cuproptosis random forest cox score (CRFCS) model

**Figure 2:**
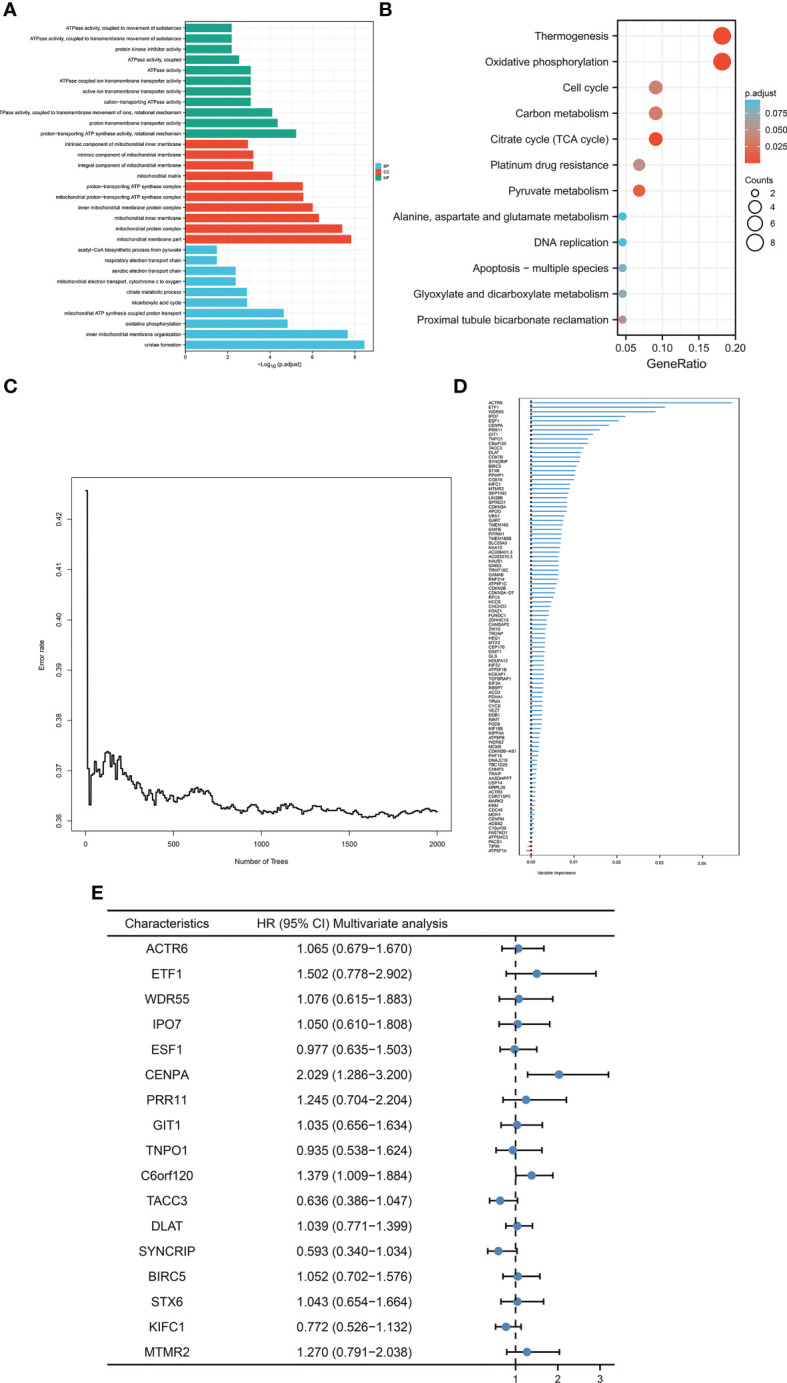
Construction of CRFCS. **(A, B)** GO **(A)** and KEGG **(B)** enrichment analysis of gene sets after generalizing features. **(C)** Trend of out-of-bag error (oob) of random forest model with the number of trees (nTree). **(D)** Ranking of variable importance (VIMP) of features. **(E)** Multivariate cox test of feature genes.

Given that cuproptosis may be involved in the progression of HCC, a more robust prognostic model was constructed using the above-mentioned cuproptosis genes with prominent prognostic significance (DLAT, PDHA1, GLS, and CDKN2A). First and foremost, correlation analysis of the above genes was initially conducted *via* analyzing data from TCGA-LIHC cohort to enhance the generalization ability of the model. For each cuproptosis gene listed above, the top 25 expression-related genes were identified as generalized features based on correlation coefficients. For the gene sets after the generalization of features, GO/KEGG analysis was performed to ensure that the characteristics were not distorted by generalization. The results indicated that the gene set after features generalization remained associated with key pathways of cuproptosis, such as the TCA cycle (**Figures 2A, B**). Training the gene set as input of the random forest model, the out-of-bag error of the model stabilized when the number of trees was approximately around 1000 (**Figure 2C**). The random forest model derived the variable importance (VIMP) ranking of the input features (**Figure 2D**). We selected the top 20% of the ranked features to be involved in the construction of the Cox model. Excluding the features not significant in the univariate Cox test, 17 features were obtained and model scores were established according to the steps in Materials and Methods (**Figure 2E**).

### 3.3 Prognosis prediction of CRFCS

**Figure 3:**
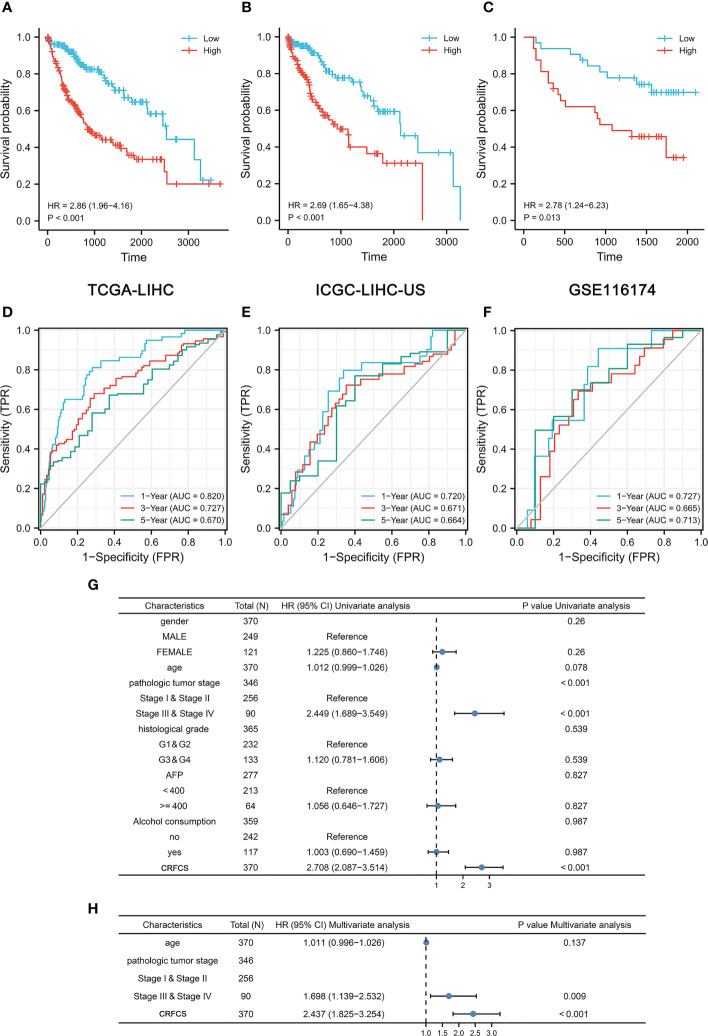
Prognosis prediction of CRFCS. **(A, C)** Kaplan-Meier curves of CRFCS subgroups for the training cohort TCGA-LIHC **(A)**, the external validation cohorts ICGC-LIHC-US **(B)**, and GSE116174 **(C)**. **(D–F)** AUC curves for the prediction of overall survival (OS) by CRFCS in samples of TCGA-LIHC **(D)**, ICGC-LIHC-US **(E)**, and GSE116174 **(F)**. **(G, H)** Univariate **(G)** and multivariate analysis **(H)** of CRFCS.

To evaluate the accuracy of the model’s predictions, we validated CRFCS in the training set TCGA-LIHC and the external validation set ICGC-LIHC-US and GSE116174. We divided the samples of each set into high and low score groups by the median of CRFCS. In the TCGA-LIHC set, the contemporaneous surviving rate of the high CRFCS subgroup samples was much lower than that of the low CRFCS subgroup. The HR for the CRFCS subgroups was 2.86 (1.96-4.16), with a p-value less than 0.001 (**Figure 3A**). Likewise, the survival of the high CRFCS subgroup samples was shorter in both validation cohorts. In the ICGC-LIHC-US cohort, the HR of the CRFCS subgroup was 2.69 (1.65-4.38) with a p-value less than 0.001 (**Figure 3B**) while the HR value was 2.78 (1.24-6.23) with a p-value of 0.013 in the GSE116174 cohort (**Figure 3C**). Subsequently, ROC analysis was performed to evaluate the diagnostic potency of CRFCS in HCC. The results demonstrated that CRFCS was a strong predictor in both training and validation cohorts (**Figures 3D–F**). The AUC values for predicting OS were 0.820 at 1 year, 0.727 at 3 years, and 0.670 at 5 years in the TCGA-LIHC training cohort (**Figure 3D**). While AUC values for predicting OS were 0.720 at 1 year, 0.671 at 3 years, and 0.664 at 5 years in the ICGC-LIHC-US cohort (**Figure 3E**) and 0.727 at 1 year, 0.665 at 3 years, and 0.713 at 5 years in the GSE116174 cohort (**Figure 3F**). Also, we performed univariate and multivariate Cox analyses of CRFCS in order to examine the potential of CRFCS as an OS-independent prognostic factor for HCC. The results showed a hazard ratio of 2.708 (2.087-3.514) for CRFCS in the univariate analysis with a p-value less than 0.001 (**Figure 3G**). In the multifactorial analysis, the hazard ratio was 2.437 (1.825-3.254) with a p-value less than 0.001 (**Figure 3H**). These results implied that CRFCS was a potential independent predictor of HCC.

### 3.4 CRFCS and mutation features

**Figure 4:**
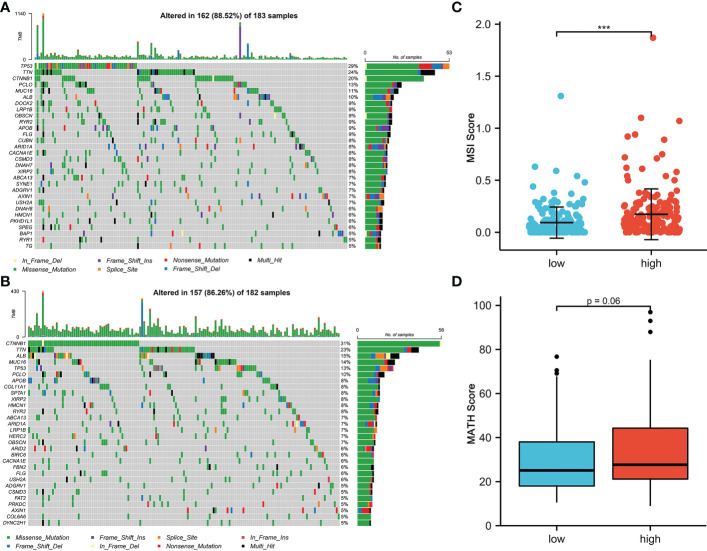
CRFCS and mutation characteristics. **(A, B)** Mutation oncoplots of high CRFCS group **(A)** and low CRFCS group **(B)**, including genes with top 30 mutation frequency. **(C)** Differences in MSI scores between high and low CRFCS subgroups. *** stands for p<0.001. **(D)** Differences in MATH scores between CRFCS subgroups.

Mutational features are an integral part of the cancer process landscape. We investigated the mutational characteristics of the CRFCS subgroup of HCC. The top 3 high-frequency mutated genes in the high-CRFCS subgroup were TP53 (29%), TTN (24%), and CTNNB1 (20%) (**Figure 4A**) while CTNNB1 (31%), TNN (23%), and ALB (15%) were identified as the top 3 mutated genes in the low CRFCS subgroup (**Figure 4B**). We also found that Microsatellite Instability (MSI) score was significantly higher in the high CRFCS subgroup than in the low CRFCS group (p<0.001) (**Figure 4C**). Then, we evaluated the MATH scores which were positively correlated with tumor heterogeneity. The results revealed that the MATH scores between the two groups were not significant (**Figure 4D**).

### 3.5 CRFCS and TME

**Figure 5:**
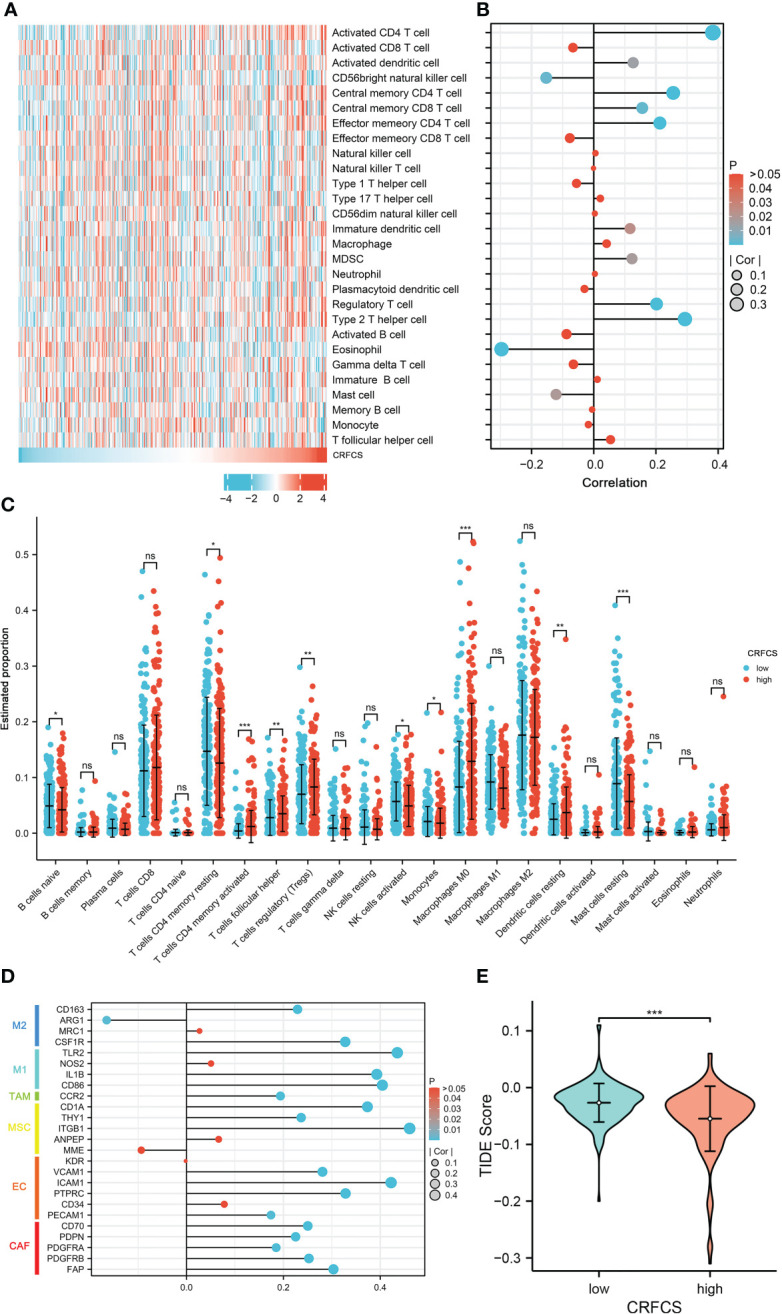
CRFCS and immune infiltration. **(A)** Heat map of ssGSEA score of various immune cells in high and low CRFCS groups via analyzing TCGA-LIHC cohort data. **(B)** The correlations between immune score and CRFCS. **(C)** Immune infiltration landscape of TCGA-LIHC cohort samples assessed by the CIBERSORT algorithm. **(D)** Correlation of stromal cell-associated markers with CRFCS. **(E)** The level of immune escape between high and low CRFCS subgroups was assessed by the TIDE algorithm. (*p<0.05; **p<0.01; ***p<0.001; ns stands for not significant).

Immunotherapy is vital for the treatment of patients with advanced cancer and TME features are essential indicators of the efficacy of immune checkpoint inhibitors (ICIs). The level of various immune-related cellular infiltrates in TCGA-LIHC cohort samples was assessed by the ssGSEA method (**Figures 5A, B**). The results displayed a positive correlation between the CRFCS and the level of some anti-tumor immune cell infiltration, such as activated CD4 T cells (p<0.0001), activated dendritic cells (p =0.0142), central memory CD4 T cells (p<0.0001), central memory CD8 T cells (p =0.0025), and effector memory CD4 T cell (p<0.0001). Similarly, infiltrations of pro-tumor immune cells including regulatory T cells (p<0.0001), type 2 T helper cells (p<0.0001), immature dendritic cells (p =0.0239), and MDSC (p =0.0173) were also positively correlated with CRFCS. In addition, some neutral immune infiltrates such as eosinophil (p<0.0001) and mast cell (p =0.0189) were negatively related to CRFCS. We also evaluated the immune infiltration of the samples with the CIBERSORT algorithm (**Figure 5C**). Higher infiltration levels of T cells CD4 memory activated (p<0.001), T cells follicular helper (p<0.01), T cells regulatory (Tregs) (p<0.01), Macrophages M0 (p<0.001) and dendritic cells resting (p<0.01) were observed in the high CRFCS subgroup. In contrast, B cells naïve (p<0.05), T cells CD4 memory resting (p<0.05), NK cells activated (p<0.05), monocytes (p<0.05) and mast cells resting (p<0.001) had higher levels in the low CRFCS subgroup. Considering both methods together, the infiltration levels of activated CD4 T cells and regulatory T cells were significantly higher in the high-CRFCS subgroup, while the infiltration level of Mast cells resting was lower. Infiltration of stromal cells is also an integral part of TME. We also assessed the levels of stromal cell-related markers in the TCGA-LIHC cohort samples. The analysis showed that the levels of most markers of diverse stromal cells including CAF, EC, MSC, TAM, M1, and M2 in the samples were positively correlated with CRFCS (**Figure 5D**). Regulatory T cells was reported to suppress the immune response and promote tumorigenic immune escape (35). We then assessed the extent of immune escape between high and low CRFCS subgroups by the TIDE algorithm and the results showed that the high CRFCS group displayed a decreased TIDE score compared with the low CRFCS group (**Figure 5E**), indicating that samples with high CRFCS had lower levels of immune escape.

### 3.6 CRFCS and drug-sensitivity

**Figure 6:**
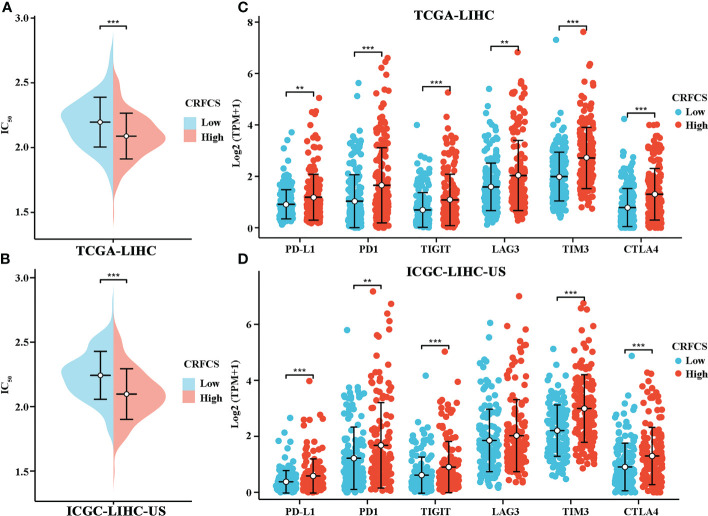
CRFCS and drug sensitivity. **(A, B)** Drug sensitivity of sorafenib in high and low CRFCS subgroups via analyzing data from TCGA cohort **(A)** and ICGC-LIHC **(B)**. **(C, D)** Differential expression of immune checkpoints between high and low CRFCS subgroups via analyzing data from TCGA cohort **(C)** and ICGC-LIHC **(D)**. (**p<0.01; ***p<0.001).

Next, we assessed the drug-sensitivity of CRFCS in HCC by applying the R package of “pRRophetic”. By analyzing data from TCGA, we found that the high CRFCS group had a lower IC_50_ of sorafenib compared with the low CRFCS group (**Figure 6A**). To verify these results, an external data from ICGC-LIHC-US was analyzed which confirmed that the high CRFCS group are more sensitive to sorafenib (**Figure 6B**). Immunotherapy delivers more opportunities to patients with advanced HCC (36). It is well recognized that TME characteristic can significantly influence the outcome of immunotherapy (37). TME is classified into three subtypes: immune-desert, immune-inflamed, and immune-excluded. The immune-inflamed type which is highly expressed with immune checkpoint such as PD1 and PD-L1 is considered to be very sensitive to immunotherapy (38). Therefore, we evaluated the expression profile of immune checkpoint in CRFCS. Our results displayed that the immune checkpoints including PD-L1, PD1, TIGIT, TIM3, and CTLA4 were significantly highly expressed in the high CRFCS group compared with the low CRFCS group in both TCGA and ICGC HCC cohorts (**Figures 6C, D**). These results suggested that high CRFCS group might be more responsive to immunotherapy.

## 4 Discussion

Cuproptosis, a recently discovered new programmed cell death induced by excessive accumulation of intracellular Cu, is distinct from known cell death forms including apoptosis, pyroptosis, ferroptosis, necrosis. To dissect the specific regulators of cuproprosis, Tsvetkov et al. used genome-wide CRISPR/Cas9 screens and identify ten crucial cuproptosis-specific genes including FDX1, LIAS, LIPT1, DLD, DLAT, PDHA1, PDHB, MTF1, GLS, and CDKN2A (25). These ten genes are closely associated with HCC progression and TME. Zhang et al. identified FDX1 as an immunotherapy predictor of HCC (39). Yan et al. discovered that inhibition of LIPT1 restrained HCC cell proliferation and invasion (40). Zhou et al. found that overexpression of DLAT increased HCC cell growth and invasion and may facilitate cancer cell evade immune system (41). Sun et al. reported that activation of PDHA1 suppressed the Warburg effect and promoted HCC apoptosis (42). Yang et al. demonstrated that knockdown PDHB induced metabolic reprogramming of the tricarboxylic acid (TCA) cycle leading to glutamine depletion and inhibition of HCC cell proliferation (43). Yang et al. reported that over-expression of MTF1 contributed to the proliferation of HCC cells (44). Dong et al. found that GLS1 promoted HCC cell proliferation *via* activating AKT/GSK3β/Cyclin D1 pathway (45). Xu et al. revealed that upregulation of CDKN2A significantly inhibited ACTR5 induced HCC cell proliferation (46). Considering the role of these ten crucial cuproptosis-specific genes in HCC, constructing a model based on these ten genes might provide potential insights for evaluation the TME and immunotherapy efficacy of HCC.

Since the discovery of cuproptosis, the role of cuptoptosis in liver cancer prognosis and TME has been gradually evaluated. Previous studies mainly explored this issue by constructing Lasso cox model, which directly entered the target genes as model inputs (47–53). The Lasso model is applied to analyze multicollinearity data (54). Usually, nonlinear data might be generated when performing log normalization of the expression matrix. From this perspective, the lasso cox model might not be the ideal strategy. The random forest model is a set of binary trees constructed with recursive partitioning (RPART), which enables the random forest to handle nonlinear data due to the combination of trees (55). Therefore, the random forest model with nonlinear data as the application object is more suitable. Meanwhile, the random forest model is better at learning potential crossover features consisting of multidimensional features (56) and shows strong robustness when applied to large feature sets (57). These reasons led us to use the random forest model to construct the prognostic model. In addition, considering that there might be noise differences between individual data of each sample, some features may be lost due to the presence of data noise if the target genes are considered only, we trained the model using gene clusters related to cuproptosis genes as model inputs to generalize the features. We generalize the features by acquiring highly correlated genes of crucial cuproptosis genes when constructing the model so that the model could learn as much information as possible about the implicit features in the data. This makes the output of the model smoother and less susceptible to fluctuations caused by noise in the data, thus improving the robustness of the model. The generalized input data combined with the random forest model can better learn the potential cross features in the data.

Microsatellite instability (MSI) is closely correlated with tumor immunotherapy efficacy. High MSI (MSI-H) in tumor samples usually cause additional mutant antigens and sensitize patient to immunotherapy (58). However, MSI-H also tends to increase tumor heterogeneity, which in turn results in poorer immunotherapy efficacy (59). In the present study, the mutation landscape of CRFCS subgroups was investigated which showed that the MSI scores were significantly higher in the high-CRFCS subgroup sample than in the low-CRFCS group while no significant difference between high and low CRFCS subgroups was observed in the tumor heterogeneity score MATH (**Figure 4**). These results suggested that high CRFCS subgroups may have better immunotherapeutic efficacy. Besides, studies exist demonstrated that tumor patients with high expression of immune checkpoints are more sensitive to immunotherapy (38). We evaluated the expression of immune checkpoints in high and low CRFCS group. Our results showed that the immune checkpoints including PD-L1, PD1, TIGIT, TIM3, and CTLA4 were remarkably highly expressed in the high CRFCS group compared with the low CRFCS group (**Figures 6C, D**). In addition, evidence displayed that Treg cells cause immune escape through several mechanisms, which in turn impede the anti-tumor immune response (60). To estimate the tumor immune escape effect between the CRFCS subgroups, we calculated the TIDE scores of the samples. The results showed that the high-CRFCS subgroup had significantly lower TIDE scores (**Figure 5E**), indicating that samples of the high-CRFCS subgroup had a lower probability of immune escape and were less prone to be resistant to immunotherapy. Combining the results above, it might be inferred that the high CRFCS group might be more suitable to receive immunotherapy than the low CRFCS group.

Although a cuproptosis related model termed CRFCS was successfully constructed to evaluate prognosis and TME characteristic in HCC, some limitations should not be neglected. First, cuproptosis was discovered in 2022, only several genes were confirmed as crucial cuproptosis-specific genes, more genes need to be identified to provide systematic and comprehensive understanding of cuproptosis. Second, our study was performed based on integrative bioinformatic analysis, it would be more valid to carry out functional experiments *in vitro* and *in vivo*. Finally, the data involved in this study were retrieved from public dataset, it would be better to use large-scale of local datasets to verify our findings.

## 5 Conclusions

In aggregate, we constructed a cuproptosis random forest cox score (CRFCS) model. CRFCS was identified to be an independent prognostic indicator of HCC and high CRFCS samples showed a poor prognosis. Interestingly, CRFCS were correlated with TME characteristics as well as clinical treatment efficacy. Patients with high CRFCS had a better clinical prognosis for immunotherapy and sorafenib.

## Data availability statement

The datasets presented in this study can be found in online repositories. The names of the repository/repositories and accession number (s) can be found below: https://portal.gdc.cancer.gov/, TCGA-LIHC; https://www.ncbi.nlm.nih.gov/geo/, GSE116174; https://dcc.icgc.org/projects/, ICGC-LIHC-US.

## Author contributions

LW and JW designed and planned the study concept. RL, YL, and FZ performed experiments and analyzed data. RL and LW drafted the manuscript. All authors contributed to the article and approved the submitted version.

## References

[1] Sung H Ferlay J Siegel RL Laversanne M Soerjomataram I Jemal A. Global cancer statistics 2020: GLOBOCAN estimates of incidence and mortality worldwide for 36 cancers in 185 countries. CA Cancer J Clin (2021) 71(3):209–49. doi: 10.3322/caac.21660 33538338

[2] Bray F Ferlay J Soerjomataram I Siegel RL Torre LA Jemal A. Global cancer statistics 2018: GLOBOCAN estimates of incidence and mortality worldwide for 36 cancers in 185 countries. CA Cancer J Clin (2018) 68(6):394–424. doi: 10.3322/caac.21492 30207593

[3] Sim HW Knox J. Hepatocellular carcinoma in the era of immunotherapy. Curr Probl Cancer (2018) 42(1):40–8. doi: 10.1016/j.currproblcancer.2017.10.007 29150141

[4] Kudo M Finn RS Qin S Han KH Ikeda K Piscaglia F. Lenvatinib versus sorafenib in first-line treatment of patients with unresectable hepatocellular carcinoma: a randomised phase 3 non-inferiority trial. Lancet (2018) 391(10126):1163–73. doi: 10.1016/S0140-6736(18)30207-1 29433850

[5] Topalian SL Hodi FS Brahmer JR Gettinger SN Smith DC McDermott DF. Safety, activity, and immune correlates of anti-PD-1 antibody in cancer. N Engl J Med (2012) 366(26):2443–54. doi: 10.1056/NEJMoa1200690 PMC3544539 22658127

[6] Nishida N Kudo M. Immunological microenvironment of hepatocellular carcinoma and its clinical implication. Oncology (2017) 92 Suppl 1:40–9. doi: 10.1159/000451015 27764823

[7] Kurebayashi Y Ojima H Tsujikawa H Kubota N Maehara J Abe Y. Landscape of immune microenvironment in hepatocellular carcinoma and its additional impact on histological and molecular classification. Hepatology (2018) 68(3):1025–41. doi: 10.1002/hep.29904 29603348

[8] Chen L Min J Wang F. Copper homeostasis and cuproptosis in health and disease. Signal Transduct Target Ther (2022) 7(1):378. doi: 10.1038/s41392-022-01229-y 36414625 PMC9681860

[9] Baltaci AK Dundar TK Aksoy F Mogulkoc R. Changes in the serum levels of trace elements before and after the operation in thyroid cancer patients. Biol Trace Elem Res (2017) 175(1):57–64. doi: 10.1007/s12011-016-0768-2 27263537

[10] Stepien M Jenab M Freisling H Becker NP Czuban M Tjønneland A. Pre-diagnostic copper and zinc biomarkers and colorectal cancer risk in the European prospective investigation into cancer and nutrition cohort. Carcinogenesis (2017) 38(7):699–707. doi: 10.1093/carcin/bgx051 28575311

[11] Zhang X Yang Q. Association between serum copper levels and lung cancer risk: A meta-analysis. J Int Med Res (2018) 46(12):4863–73. doi: 10.1177/0300060518798507 PMC6300955 30296873

[12] Saleh SAK Adly HM Abdelkhaliq AA Nassir AM. Serum levels of selenium, zinc, copper, manganese, and iron in prostate cancer patients. Curr Urol (2020) 14(1):44–9. doi: 10.1159/000499261 PMC7206590 32398996

[13] Ge EJ Bush AI Casini A Cobine PA Cross JR DeNicola GM. Connecting copper and cancer: from transition metal signalling to metalloplasia. Nat Rev Cancer (2022) 22(2):102–13. doi: 10.1038/s41568-021-00417-2 PMC8810673 34764459

[14] Li Y. Copper homeostasis: Emerging target for cancer treatment. IUBMB Life (2020) 72(9):1900–8. doi: 10.1002/iub.2341 32599675

[15] Vanderwerf SM Cooper MJ Stetsenko IV Lutsenko S. Copper specifically regulates intracellular phosphorylation of the wilson's disease protein, a human copper-transporting ATPase. J Biol Chem (2001) 276(39):36289–94. doi: 10.1074/jbc.M102055200 11470780

[16] Tsang T Posimo JM Gudiel AA Cicchini M Feldser DM Brady DC. Copper is an essential regulator of the autophagic kinases ULK1/2 to drive lung adenocarcinoma. Nat Cell Biol (2020) 22(4):412–24. doi: 10.1038/s41556-020-0481-4 PMC7610258 32203415

[17] Cui L Gouw AM LaGory EL Guo S Attarwala N Tang Y. Mitochondrial copper depletion suppresses triple-negative breast cancer in mice. Nat Biotechnol (2021) 39(3):357–67. doi: 10.1038/s41587-020-0707-9 PMC7956242 33077961

[18] Brady DC Crowe MS Greenberg DN Counter CM. Copper chelation inhibits BRAFV600E-driven melanomagenesis and counters resistance to BRAFV600E and MEK1/2 inhibitors. Cancer Res (2017) 77(22):6240–52. doi: 10.1158/0008-5472.CAN-16-1190 PMC5690876 28986383

[19] O'Dell BL. Interleukin-2 production is altered by copper deficiency. Nutr Rev (1993) 51(10):307–9. doi: 10.1111/j.1753-4887.1993.tb03062.x 8302489

[20] Prohaska JR Lukasewycz OA. Copper deficiency suppresses the immune response of mice. Science (1981) 213(4507):559–61. doi: 10.1126/science.7244654 7244654

[21] Jones DG. Effects of dietary copper depletion on acute and delayed inflammatory responses in mice. Res Vet Sci (1984) 37(2):205–10. doi: 10.1016/S0034-5288(18)31906-4 6505401

[22] Voli F Valli E Lerra L Kimpton K Saletta F Giorgi FM. Intratumoral copper modulates PD-L1 expression and influences tumor immune evasion. Cancer Res (2020) 80(19):4129–44. doi: 10.1158/0008-5472.CAN-20-0471 32816860

[23] Shanbhag VC Gudekar N Jasmer K Papageorgiou C Singh K Petris MJ. Copper metabolism as a unique vulnerability in cancer. Biochim Biophys Acta Mol Cell Res (2021) 1868(2):118893. doi: 10.1016/j.bbamcr.2020.118893 33091507 PMC7779655

[24] Denoyer D Masaldan S La Fontaine S Cater MA. Targeting copper in cancer therapy: 'Copper that cancer'. Metallomics (2015) 7(11):1459–76. doi: 10.1039/c5mt00149h 26313539

[25] Tsvetkov P Coy S Petrova B Dreishpoon M Verma A Abdusamad M. Copper induces cell death by targeting lipoylated TCA cycle proteins. Science (2022) 375(6586):1254–61. doi: 10.1126/science.abf0529 PMC9273333 35298263

[26] Zhu Z Zhao Q Song W Weng J Li S Guo T. A novel cuproptosis-related molecular pattern and its tumor microenvironment characterization in colorectal cancer. Front Immunol (2022) 13:940774. doi: 10.3389/fimmu.2022.940774 36248908 PMC9561547

[27] Li W Zhang X Chen Y Pang D. Identification of cuproptosis-related patterns and construction of a scoring system for predicting prognosis, tumor microenvironment-infiltration characteristics, and immunotherapy efficacy in breast cancer. Front Oncol (2022) 12:966511. doi: 10.3389/fonc.2022.966511 36212436 PMC9544817

[28] Shen Y Li D Liang Q Yang M Pan Y Li H. Cross-talk between cuproptosis and ferroptosis regulators defines the tumor microenvironment for the prediction of prognosis and therapies in lung adenocarcinoma. Front Immunol (2023) 13:1029092. doi: 10.3389/fimmu.2022.1029092 36733399 PMC9887127

[29] Song Q Zhou R Shu F Fu W. Cuproptosis scoring system to predict the clinical outcome and immune response in bladder cancer. Front Immunol (2022) 13:958368. doi: 10.3389/fimmu.2022.958368 35990642 PMC9386055

[30] Cai Z He Y Yu Z Hu J Xiao Z Zu X. Cuproptosis-related modification patterns depict the tumor microenvironment, precision immunotherapy, and prognosis of kidney renal clear cell carcinoma. Front Immunol (2022) 13:933241. doi: 10.3389/fimmu.2022.933241 36211378 PMC9540508

[31] Mroz EA Rocco JW. MATH, a novel measure of intratumor genetic heterogeneity, is high in poor-outcome classes of head and neck squamous cell carcinoma. Oral Oncol (2013) 49(3):211–5. doi: 10.1016/j.oraloncology.2012.09.007 PMC3570658 23079694

[32] Niu B Ye K Zhang Q Lu C Xie M McLellan MD. MSIsensor: microsatellite instability detection using paired tumor-normal sequence data. Bioinformatics (2014) 30(7):1015–6. doi: 10.1093/bioinformatics/btt755 PMC3967115 24371154

[33] Charoentong P Finotello F Angelova M Mayer C Efremova M Rieder D. Pan-cancer immunogenomic analyses reveal genotype-immunophenotype relationships and predictors of response to checkpoint blockade. Cell Rep (2017) 18(1):248–62. doi: 10.1016/j.celrep.2016.12.019 28052254

[34] Jiang P Gu S Pan D Fu J Sahu A Hu X. Signatures of T cell dysfunction and exclusion predict cancer immunotherapy response. Nat Med (2018) 24(10):1550–8. doi: 10.1038/s41591-018-0136-1 PMC6487502 30127393

[35] Yin Y Cai X Chen X Liang H Zhang Y Li J. Tumor-secreted miR-214 induces regulatory T cells: A major link between immune evasion and tumor growth. Cell Res (2014) 24(10):1164–80. doi: 10.1038/cr.2014.121 PMC4185347 25223704

[36] Hato T Goyal L Greten TF Duda DG Zhu AX. Immune checkpoint blockade in hepatocellular carcinoma: Current progress and future directions. Hepatology (2014) 60(5):1776–82. doi: 10.1002/hep.27246 PMC4211962 24912948

[37] Pardoll DM. The blockade of immune checkpoints in cancer immunotherapy. Nat Rev Cancer (2012) 12(4):252–64. doi: 10.1038/nrc3239 PMC4856023 22437870

[38] Doroshow DB Bhalla S Beasley MB Sholl LM Kerr KM Gnjatic S. PD-L1 as a biomarker of response to immune-checkpoint inhibitors. Nat Rev Clin Oncol (2021) 18(6):345–62. doi: 10.1038/s41571-021-00473-5 33580222

[39] Zhang C Zeng Y Guo X Shen H Zhang J Wang K. Pan-cancer analyses confirmed the cuproptosis-related gene FDX1 as an immunotherapy predictor and prognostic biomarker. Front Genet (2022) 13:923737. doi: 10.3389/fgene.2022.923737 35991547 PMC9388757

[40] Yan C Niu Y Ma L Tian L Ma J. System analysis based on the cuproptosis-related genes identifies LIPT1 as a novel therapy target for liver hepatocellular carcinoma. J Transl Med (2022) 20(1):452. doi: 10.1186/s12967-022-03630-1 36195876 PMC9531858

[41] Zhou Y Gu H Shao B Zhang S Pall H Peixoto RD. Glycolysis-related gene dihydrolipoamide acetyltransferase promotes poor prognosis in hepatocellular carcinoma through the wnt/β-catenin and PI3K/Akt signaling pathways. Ann Transl Med (2022) 10(22):1240. doi: 10.21037/atm-22-5272 36544660 PMC9761179

[42] Sun J Li J Guo Z Sun L Juan C Zhou Y. Overexpression of pyruvate dehydrogenase E1α subunit inhibits warburg effect and induces cell apoptosis through mitochondria-mediated pathway in hepatocellular carcinoma. Oncol Res (2019) 27(4):407–14. doi: 10.3727/096504018X15180451872087 PMC7848459 29444744

[43] Yang C Lee D Zhang MS Tse AP Wei L Bao MH. Genome-wide CRISPR/Cas9 library screening revealed dietary restriction of glutamine in combination with inhibition of pyruvate metabolism as effective liver cancer treatment. Adv Sci (Weinh) (2022) 9(34):e2202104. doi: 10.1002/advs.202202104 36310121 PMC9731711

[44] Yang Y Qian Cai Q Sheng Fu L Wei Dong Y Fan F Zhong Wu X. Reduced N6-methyladenosine mediated by METTL3 acetylation promotes MTF1 expression and hepatocellular carcinoma cell growth. Chem Biodivers (2022) 19(11):e202200333. doi: 10.1002/cbdv.202200333 36149370

[45] Xi J Sun Y Zhang M Fa Z Wan Y Min Z. GLS1 promotes proliferation in hepatocellular carcinoma cells *via* AKT/GSK3β/CyclinD1 pathway. Exp Cell Res (2019) 381(1):1–9. doi: 10.1016/j.yexcr.2019.04.005 31054856

[46] Xu X Chan AKN Li M Liu Q Mattson N Pangeni Pokharel S. ACTR5 controls CDKN2A and tumor progression in an INO80-independent manner. Sci Adv (2022) 8(51):eadc8911. doi: 10.1126/sciadv.adc8911 36563143 PMC9788768

[47] Liu Z Qi Y Wang H Zhang Q Wu Z Wu W. Risk model of hepatocellular carcinoma based on cuproptosis-related genes. Front Genet (2022) 13:1000652. doi: 10.3389/fgene.2022.1000652 36186455 PMC9521278

[48] Xie Y Zhang W Sun J Sun L Meng F Yu H. A novel cuproptosis-related immune checkpoint gene signature identification and experimental validation in hepatocellular carcinoma. Sci Rep (2022) 12(1):18514. doi: 10.1038/s41598-022-22962-y 36323801 PMC9630496

[49] Zhang G Sun J Zhang X. A novel cuproptosis-related LncRNA signature to predict prognosis in hepatocellular carcinoma. Sci Rep (2022) 12(1):11325. doi: 10.1038/s41598-022-15251-1 35790864 PMC9256635

[50] Wang G Xiao R Zhao S Sun L Guo J Li W. Cuproptosis regulator-mediated patterns associated with immune infilltration features and construction of cuproptosis-related signatures to guide immunotherapy. Front Immunol (2022) 13:945516. doi: 10.3389/fimmu.2022.945516 36248857 PMC9559227

[51] Cong T Luo Y Liu Y Yang C Yang H Li Y. Cuproptosis-related immune checkpoint gene signature: Prediction of prognosis and immune response for hepatocellular carcinoma. Front Genet (2022) 13:1000997. doi: 10.3389/fgene.2022.1000997 36276933 PMC9579294

[52] Zhang Z Zeng X Wu Y Liu Y Zhang X Song Z. Cuproptosis-related risk score predicts prognosis and characterizes the tumor microenvironment in hepatocellular carcinoma. Front Immunol (2022) 13:925618. doi: 10.3389/fimmu.2022.925618 35898502 PMC9311491

[53] Zhou Z Zhou Y Liu D Yang Q Tang M Liu W. Prognostic and immune correlation evaluation of a novel cuproptosis-related genes signature in hepatocellular carcinoma. Front Pharmacol (2022) 13:1074123. doi: 10.3389/fphar.2022.1074123 36588699 PMC9795230

[54] Tibshirani R. Regression shrinkage and selection *Via* the lasso. J R Stat Society: Ser B (Methodological) (1996) 58:267–88. doi: 10.1111/j.2517-6161.1996.tb02080.x

[55] Breiman L. Random forests. Mach Learn (2001) 45(1):5–32. doi: 10.1023/A:1010933404324

[56] Díaz-Uriarte R Alvarez de Andrés S. Gene selection and classification of microarray data using random forest. BMC Bioinf (2006) 7:3. doi: 10.1186/1471-2105-7-3 PMC1363357 16398926

[57] Hua J Xiong Z Lowey J Suh E Dougherty ER. Optimal number of features as a function of sample size for various classification rules. Bioinformatics (2005) 21(8):1509–15. doi: 10.1093/bioinformatics/bti171 15572470

[58] Le DT Durham JN Smith KN Wang H Bartlett BR Aulakh LK. Mismatch repair deficiency predicts response of solid tumors to PD-1 blockade. Science (2017) 357(6349):409–13. doi: 10.1126/science.aan6733 PMC5576142 28596308

[59] Trinh A Polyak K. Tumor neoantigens: When too much of a good thing is bad. Cancer Cell (2019) 36(5):466–7. doi: 10.1016/j.ccell.2019.10.009 31715129

[60] Nishikawa H Koyama S. Mechanisms of regulatory T cell infiltration in tumors: implications for innovative immune precision therapies. J Immunother Cancer (2021) 9(7):e002591. doi: 10.1136/jitc-2021-002591 34330764 PMC8327843

